# Acute *Ascaris* infection impairs the effector functions of natural killer cells in single and *Salmonella* co-infected pigs

**DOI:** 10.1038/s41598-024-64497-4

**Published:** 2024-06-25

**Authors:** Robert M. Mugo, Larissa Oser, Ankur Midha, Joshua Adjah, Arkadi Kundik, Alexandra Laubschat, Philipp Höfler, Zaneta D. Musimbi, Rima Hayani, Josephine Schlosser-Brandenburg, Susanne Hartmann, Sebastian Rausch

**Affiliations:** Institute of Immunology, Centre for Infection Medicine, Freie Universität Berlin, Berlin, Germany

**Keywords:** *Ascaris*, *Salmonella*, Natural killer cells, Coinfection, Parasitic infection, Bacterial infection

## Abstract

Natural killer (NK) cells play a key role in defense against *Salmonella* infections during the early phase of infection. Our previous work showed that the excretory/secretory products of *Ascaris suum* repressed NK activity in vitro. Here, we asked if NK cell functionality was influenced in domestic pigs during coinfection with *Ascaris* and *Salmonella enterica* serotype Typhimurium. *Ascaris* coinfection completely abolished the IL-12 and IL-18 driven elevation of IFN-γ production seen in CD16 + CD8α + perforin + NK cells of *Salmonella* single-infected pigs. Furthermore, *Ascaris* coinfection prohibited the *Salmonella*-driven rise in NK perforin levels and CD107a surface expression. In line with impaired effector functions, NK cells from *Ascaris*-single and coinfected pigs displayed elevated expression of the inhibitory KLRA1 and NKG2A receptors genes, contrasting with the higher expression of the activating NKp46 and NKp30 receptors in NK cells during *Salmonella* single infection. These differences were accompanied by the highly significant upregulation of T-bet protein expression in NK cells from *Ascaris*-single and *Ascaris*/*Salmonella* coinfected pigs. Together, our data strongly indicate a profound repression of NK functionality by an *Ascaris* infection which may hinder infected individuals from adequately responding to a concurrent bacterial infection.

## Introduction

Close to a quarter of the global human population is infected with *Ascaris lumbricoides,* a soil-transmitted helminth^1,2^. Young children are especially vulnerable to high-intensity *Ascaris* infection and chronic ascariasis which is linked to malnutrition and impaired physical and cognitive development^3,4^. A closely related species, *Ascaris suum,* is widely spread in pig farming and is frequently associated with economic losses through liver condemnation in slaughterhouses and through poor feed conversion ratio^5,6^. As a result of its widespread distribution, ascariasis is associated with a myriad of microbial co-infections such as *Salmonella enterica* both in humans and pigs^7^.

Infection with the facultative intracellular *Salmonella enterica* serovar Typhimurium is one of the most common foodborne diseases in humans^8^. Domestic pigs primarily act as asymptomatic carriers and natural hosts for both zoonotic *Salmonella enterica* and *Ascaris suum* infections. In addition, domestic pigs display similar physiology and a closely related organization and composition of the immune cell repertoire compared to humans^9^. Hence, the domestic pig provides a valuable translational animal model for studying immune responses to *Ascaris* and *Salmonella*-single infections as well as coinfections.

*Ascaris*-associated immune responses are marked by a type 2 (Th2) immune response characterized by elevated levels of interleukin 4 (IL-4), IL-5, and IL-13, whereby higher Th2 cytokine production predicts lower reinfection rates in humans^10^. In addition, *Ascaris* induces an immunomodulatory environment through the induction of regulatory T cells (Treg) which are thought to partake in the elevated production of IL-10 and transforming growth factor-β (TGF-β) thereby promoting host tolerance. Together with the induction of alternatively activated macrophages, the type 2/immunomodulatory environment is geared towards the limitation of adult worm burdens and promoting wound healing, the latter being essential during larval tissue migration through the liver and lungs^7,11^. This profile is in sharp contrast to the immune responses induced in *Salmonella* infection where microbial control is highly dependent on type 1 (Th1) immunity, mainly marked by the production of interferon-gamma (IFN-γ), tumor necrosis factor-alpha (TNF-α) and IL-12^12,13^.

In accordance with the cross-regulation between opposing programs of effector cell differentiation and function, earlier work in mice clearly showed that nematode-induced Th2 responses resulted in the impaired Th1-dependent control of coinfections with *Salmonella* and with other enteric microbes^14^. It is therefore conceivable that the immune environment induced by *Ascaris* infection similarly antagonizes the host's protective cellular immune responses against *Salmonella* infection*.* To date, however, it is not clear if *Ascaris* infection entails a higher risk for systemic salmonellosis in pigs and humans. Similarly, studies investigating the potential impact of *Ascaris* coinfection on the zoonotic potential of pig meat products are largely lacking. We therefore recently investigated the consequences of *Ascaris* coinfection for the control of acute *salmonella* infection and found that coinfected pigs displayed significantly higher live *Salmonella* burdens^15^.

Natural killer (NK) cells play a central role in immunity to *Salmonella* infections through the production of pro-inflammatory cytokines such as IFN-γ and TNF-α as well as by the release of granzyme B and perforin cytotoxic effector molecules leading to the killing of the infected host cells^16,17^. Porcine NK cells general activity was shown to be diminished in the presence of IL-4 and IL-2 in vitro^18^. Furthermore, we have previously demonstrated the suppression of IFN-γ production by NK cells following in vitro stimulation with IL-12 and IL-18 in the presence of adult *Ascaris suum* excretory-secretory products^19^. In the current study, we therefore aimed to further explore the impact of an *Ascaris suum* infection on NK cell functionality in an *Ascaris* and *Salmonella* coinfection setting in domestic pigs. We show that the phenotype and function of porcine NK cells is systemically altered within two weeks of *Ascaris*-single infection and *Salmonella* coinfection, evident in diminished IL-12/IL-18-induced IFN-γ production, lower cellular degranulation capacity, and the upregulation of T-bet and inhibitory NK cell receptors.

## Results

### *Salmonella*-single infection is associated with a reduction in systemic NK cell frequencies

**Figure 1:**
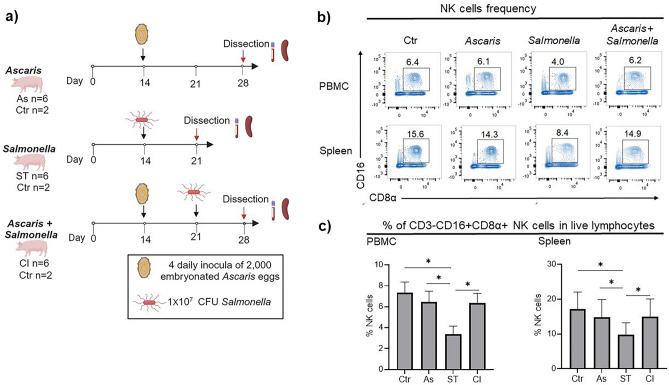
Lower frequencies of NK cells in *Salmonella-*single infection. (**a**) Experimental layout of single and coinfection with *Ascaris suum* and *Salmonella enterica*. Each infection group comprised 6 pigs. 2 uninfected controls were included per dissection day. (**b**) Representative flow cytometry contour plots showing NK cells identified as CD16 + CD8α + cells within live FSC-A/SSC-A^low^CD3- cells from peripheral blood and spleen on the day of dissection. (**c**) Frequencies (Mean + SD) of live NK cells in PBMC and spleen cells, n = 6 per group, controls (Ctr), *Ascaris* (As), *Salmonella* (ST), and coinfection (CI). Significant mean differences between the groups are indicated (ANOVA test, *p = 0.01).

To evaluate the impact of *A. suum* on NK cells during *Ascaris*-single infection and *Salmonella* coinfection, we used the domestic pig as a human-relevant model^9^. At day 7 post-*Ascaris* infection, by which time the 3^rd^ and 4^th^ larval stage of *Ascaris* undergoes hepatotracheal migration, the pigs were orally coinfected with *Salmonella enterica* serovar *Typhimurium* (Fig. 1a). Since antibodies against pan-NK markers are lacking for the pig, we adapted the gating strategy published by Gerner^20^ and Mair et al.^21^ and defined NK cells in PBMC and spleen cell lymphocytes as FSC/SSC^low^ CD3-CD16 + CD8α + cells (Fig. 1b & Supplementary Fig. 1). The gated population homogenously expressed perforin, a hallmark of NK cells^22,23^ (Supplementary Fig. 1). Notably, NK cell frequencies were significantly lower (*p* < 0.01) systemically in both blood and spleen of the *Salmonella* single-infected group compared to uninfected controls, the latter displaying the expected frequencies of NK cells of healthy pigs^21^. In contrast, NK cell frequencies were unaffected in *Ascaris*-single infection compared to the uninfected control. Interestingly, *Ascaris-Salmonella* coinfection appeared to counteract the *Salmonella*-associated decline in systemic NK cell frequencies (Fig. 1b,c). Therefore, we asked whether *Ascaris*-single and *Ascaris*–*Salmonella* coinfection might be associated with qualitative rather than quantitative changes in NK cells and further assessed NK cell functionality upon ex vivo stimulation with cytokines.

### Impaired cytokine production by NK cells during *Ascaris*-single and *Ascaris*-*Salmonella* coinfection

**Figure 2:**
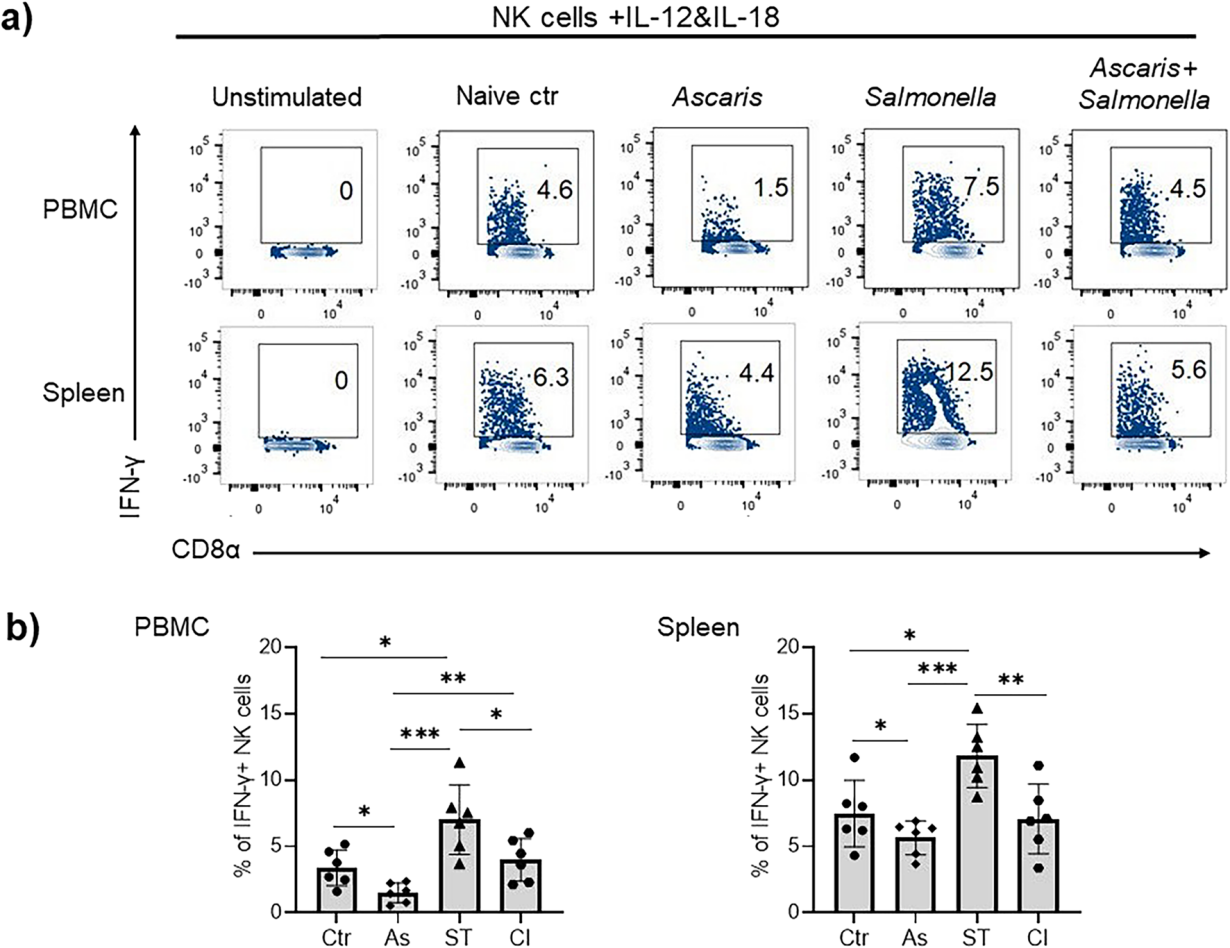
Reduced cytokine production by NK cells in *Ascaris-Salmonella* coinfection. (**a**) Exemplary flow cytometry plots of the analysis of the intracellular production of IFN-γ positive FSC/SSC^low^ CD3-CD16 + CD8α + Perforin + NK cells in PBMC and splenocyte cultures following stimulation by recombinant porcine IL-12 and IL-18. (**b**) Percentage of IFN-γ positive FSC/SSC^low^ CD3-CD16 + CD8α + Perforin + NK cells in PBMC and spleen (n = 6 per group), controls (Ctr), *Ascaris* (As), *Salmonella* (ST) and coinfection (CI). Significant mean differences between the groups are indicated (ANOVA test, *p < 0.05, **p < 0.01, ***p < 0.001).

Synergistic effects of IL-12 and IL-18 are required for NK cell activation to facilitate inflammation and pathogen elimination^16,24^. A cardinal function of NK cells is the release of copious amounts of IFN-γ in response to IL-12 and IL-18 receptor signaling, which facilitates the differentiation/expansion of Th1 cells next to promoting phagocyte effector functions^25^. To assess the potential impact of *Ascaris* infection on NK cell effector functions, we stimulated PBMC and splenocytes with IL-12/IL-18 and quantified the ensuing IFN-γ and TNF-α responses by NK cells via flow cytometry. The CD8α^dim^ NK cells population in both blood and spleen were the prominent IFN-γ producers (Fig. 2a). In sharp contrast to the lower NK cell frequencies seen in blood and spleen (Fig. 1), NK cells in the *Salmonella*-group showed a striking ability to produce IFN-γ (Fig. 2a,b) (p < 0.0001). Contrasting the unchanged NK cell frequencies, NK cells present in the blood and spleen of *Ascaris*-single infected pigs displayed significantly impaired IFN-γ responses to IL-12/18 stimulation (*p* < 0.01) (Fig. 2a,b). A similar effect was seen comparing the *Ascaris*–*Salmonella* coinfected group to *Salmonella*-single infected pigs, whereby NK cells of the latter displayed the most vigorous IFN-γ expression upon cytokine stimulation. Though in limited amounts, TNF-α production by NK cells was only evident in *Salmonella*-single infection both in blood and spleen (Supplementary Fig. 2a). Taken together, these results show that *Ascaris* suppresses IFN-γ production by NK cells during single infection, but also in pigs concurrently infected with *Ascaris* and *Salmonella* in response to IL-12 and IL-18 stimulation.

### *Ascaris* impairs the cytolytic/degranulation potential of NK cells in *Ascaris*–*Salmonella* coinfection

**Figure 3:**
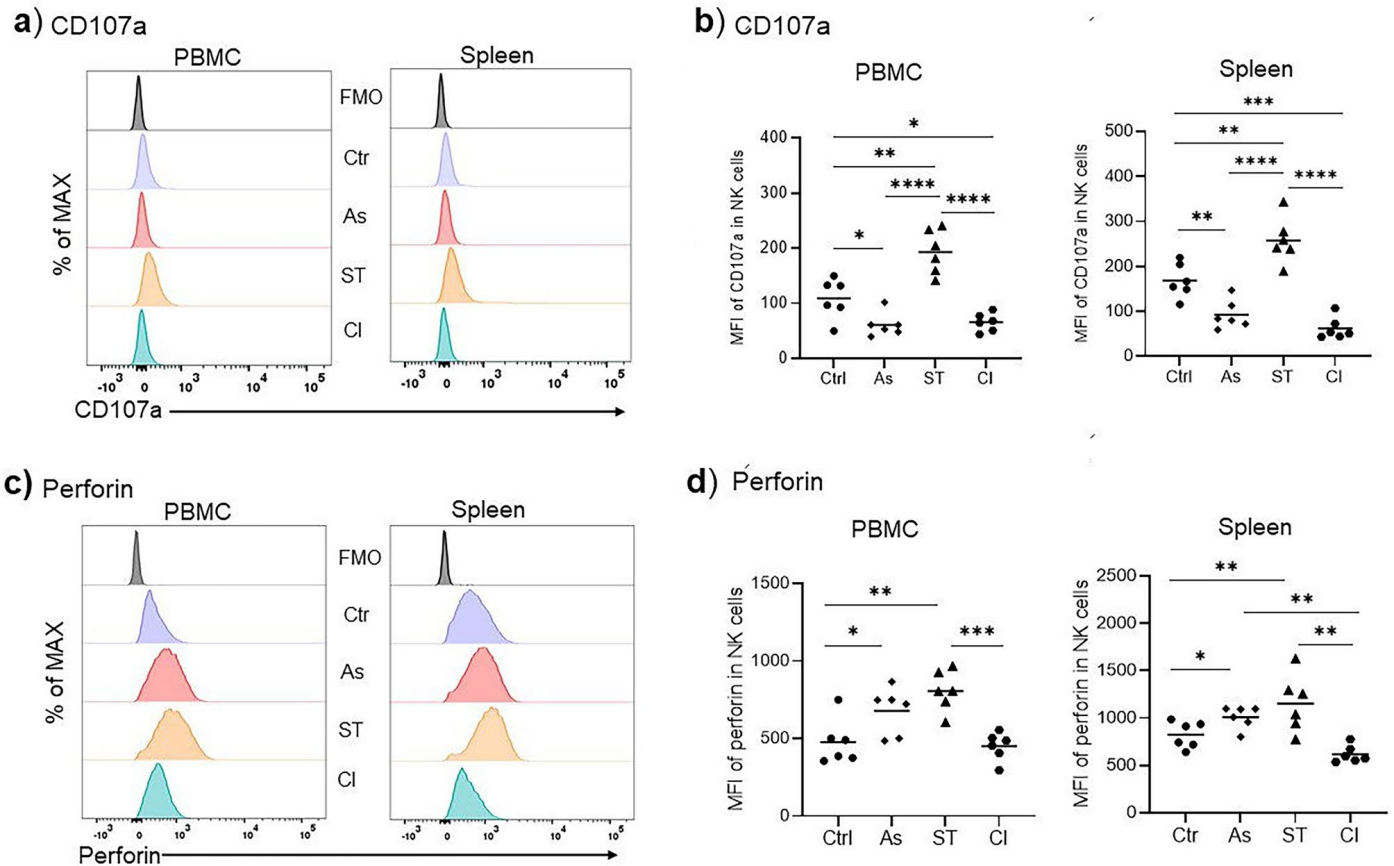
Impaired cytolytic potential of FSC/SSC^low^ CD3-CD16 + CD8α + Perforin + NK cells in *Ascaris-Salmonella* coinfection. (**a**) Representative histograms of the mean fluorescence intensity of CD107a in PBMC and spleen porcine NK cells of the control, *Ascaris-*infected, and *Ascaris-Salmonella* coinfected pigs following stimulation with recombinant IL-12 and IL-18 cytokines. (**b**) Comparisons of the CD107a mean fluorescence intensity of blood and splenic NK cells (n = 6 for all groups). (**c**) Representative histograms of the mean fluorescence intensity of perforin in PBMC and spleen NK cells. (**d**) Comparisons of the perforin mean fluorescence intensity of blood and spleen NK cells (n = 6 for all groups). Controls (Ctr), *Ascaris* (As), *Salmonella* (ST), and coinfection (CI). Significant mean differences between the groups are indicated (ANOVA test, *p < 0.05, **p < 0.01, ***p < 0.001, ****p < 0.0001).

IL-12 and IL-18 produced by *Salmonella*-infected macrophages and dendritic cells are essential prerequisites to trigger NK cell degranulation^16^. The expression of the lysosomal protein CD107a (Lysosome-Associated Membrane Protein 1, LAMP-1) is correlated with NK cell cytotoxicity^26^. Therefore, we evaluated NK cells surface expression of CD107a following stimulation with IL-12 and IL-18 cytokines. Interestingly, *Ascaris* significantly suppressed the expression of the degranulation marker CD107a compared to the controls (p < 0.01) both in blood and spleen. The NK cell degranulation repression was also evident in the coinfected group compared to the controls (p < 0.01) and *Salmonella*-single infected group (p < 0.0001) (Fig. 3a,b).

IL-18 can also prime NK cells’ cytotoxicity thus enhancing the release of perforin which forms pores on the infected cells facilitating the uptake of granzymes which induces cell lysis or apoptotic death^27,28^. In agreement with the higher expression of the degranulation marker CD107a, perforin was also upregulated in the *Salmonella*-single infected group (Fig. 3c). This increase in perforin expression was inhibited in the *Ascaris-Salmonella* coinfection setting (Fig. 3c,d). In contrast, the *Ascaris*-single infected group differed from the *Ascaris-Salmonella* coinfected group as there was a significant upregulation of perforin in the *Ascaris*-group compared to the controls. The higher CD107a expression by the splenic NK cells of the *Salmonella* single-infected group was associated with significantly higher levels of CD8α expression (p < 0.001) compared to the coinfected group (Supplementary Fig. 2b). This upregulation of CD8α was not evident in the peripheral blood NK cells. In addition, blood and splenic NK cells of the *Salmonella*-single infected pigs showed a two-fold increase in the percentages of the CD107a + NK cells compared to *Ascaris-*single and *Ascaris-Salmonella* groups (Supplementary Fig. 3a,b). These results suggest that NK cell cytotoxicity is inhibited during an acute *Ascaris* infection and in coinfection with *Ascaris* and *Salmonella* enteric microbes. Notably, systemic NK cell numbers are low in *Salmonella* single-infected pigs (Fig. 1), but the NK population is enriched in cells displaying high responsiveness to cytokine signals as well as elevated expression of degranulation markers. The decline in systemic NK cell frequencies might be partially explained by the extensive expression of NK effector functions and subsequent cell death in *Salmonella* single-infected pigs, as we determined moderately higher rates of NK cells marked by viability dyes in flow cytometry analyses of samples from this group.

### Reduced functionality of NK cells in *Ascaris*–*Salmonella* coinfection is associated with upregulation of T-bet transcription factor

**Figure 4:**
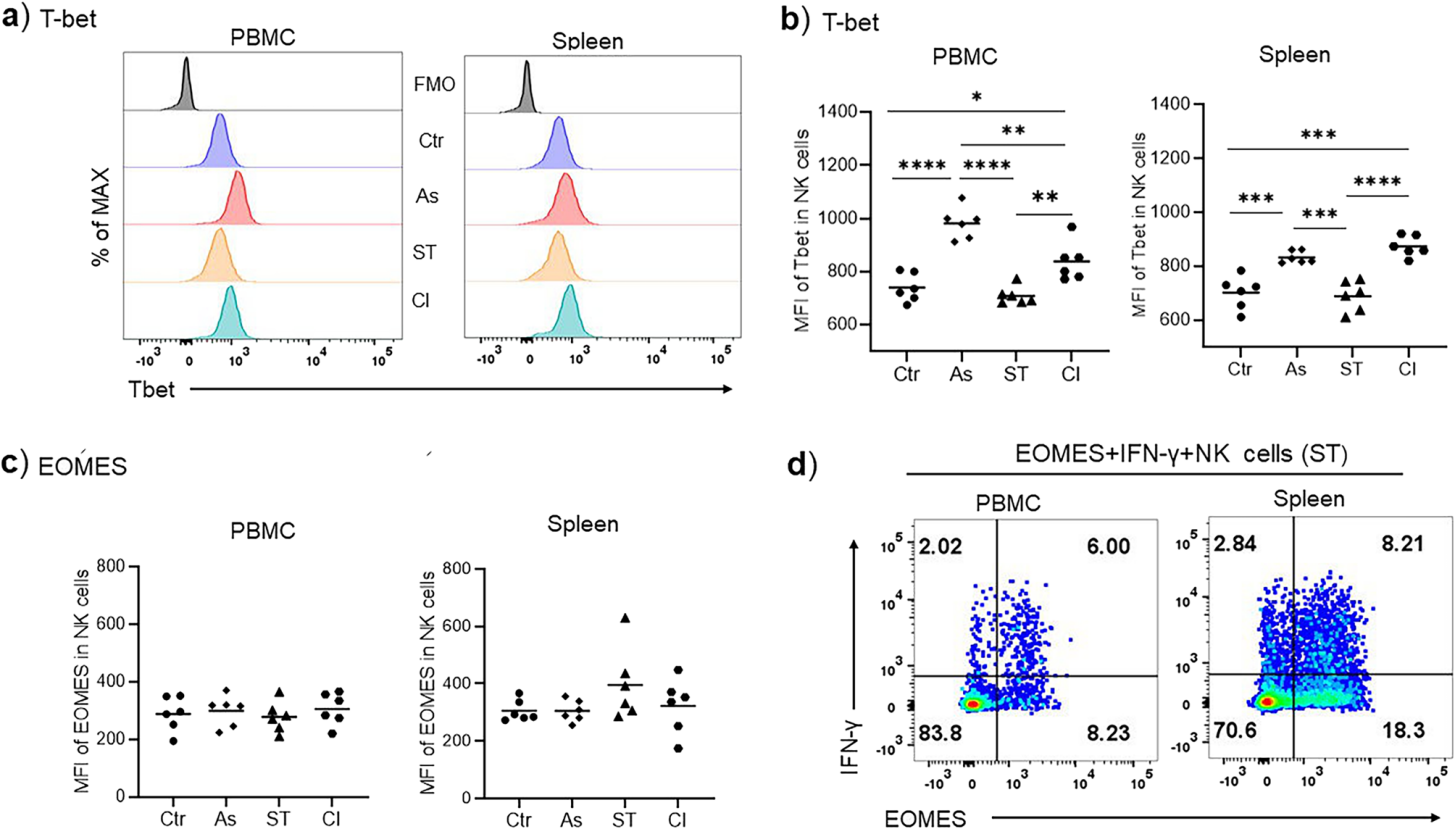
*Ascaris* induces upregulation of T-bet transcription factors in FSC/SSC^low^ CD3-CD16 + CD8α + Perforin + NK cells during *Ascaris-Salmonella* coinfection. (**a**) Representative histograms of the mean fluorescence intensity of T-bet in PBMC and spleen NK cells. (**b**) Comparisons of the T-bet mean fluorescence intensity of blood and spleen NK cells (n = 6 for all groups). (**c**) Comparisons of the EOMES mean fluorescence intensity of blood and spleen NK cells (n = 6 for all groups). (**d**) Representative flow cytometry pseudocolor plots of the EOMES + IFNγ + of the FSC/SSC^low^ CD3-CD16 + CD8α + Perforin + NK cells from the *Salmonella* group. Controls (Ctr), *Ascaris* (As), *Salmonella* (ST), and coinfection (CI). Significant mean differences between the groups are indicated (ANOVA test, *p < 0.05, **p < 0.01, ***p < 0.001, ****p < 0.0001).

We next investigated the NK cell expression of the transcription factors Eomesodermin (EOMES) and T-bet which are the master regulators of NK cell maturation, differentiation and functionality^29^. T-bet is key for NK cells’ terminal differentiation and induction of the inhibitory receptors^30^. EOMES is mainly expressed by immature NK cells though they balance their expression with T-bet to facilitate NK cells maturation. EOMES-deficient NK cells exhibit poor cytotoxicity, whereas T-bet deficient NK cells possess a normal cytotoxicity functionality^31^. We therefore evaluated the associations between the balance of these transcription factors and the functionality differences observed above. Strikingly, NK cells from *Ascaris*-infected pigs were marked by the highly significant upregulation of T-bet protein expression levels (p < 0.0001) compared with the uninfected controls (Fig. 4a,b). In line with the functional differences observed above, significant upregulation of T-bet transcription factors (p < 0.0001) in the *Ascaris-Salmonella* group compared to the control and *Salmonella*-single groups was noticed. Conversely, there were no significant changes in EOMES expression between the groups (Fig. 4c). However, EOMES-positive NK cells were prominent in IFN-γ production (Fig. 4d). Together, these data indicate that *Ascaris* modulates porcine NK cells through upregulation of T-bet possibly interfering with NK cell maturation and terminal differentiation.

### *Ascaris* modulation of NK cells is associated with upregulation of natural killer inhibitory receptors

**Figure 5:**
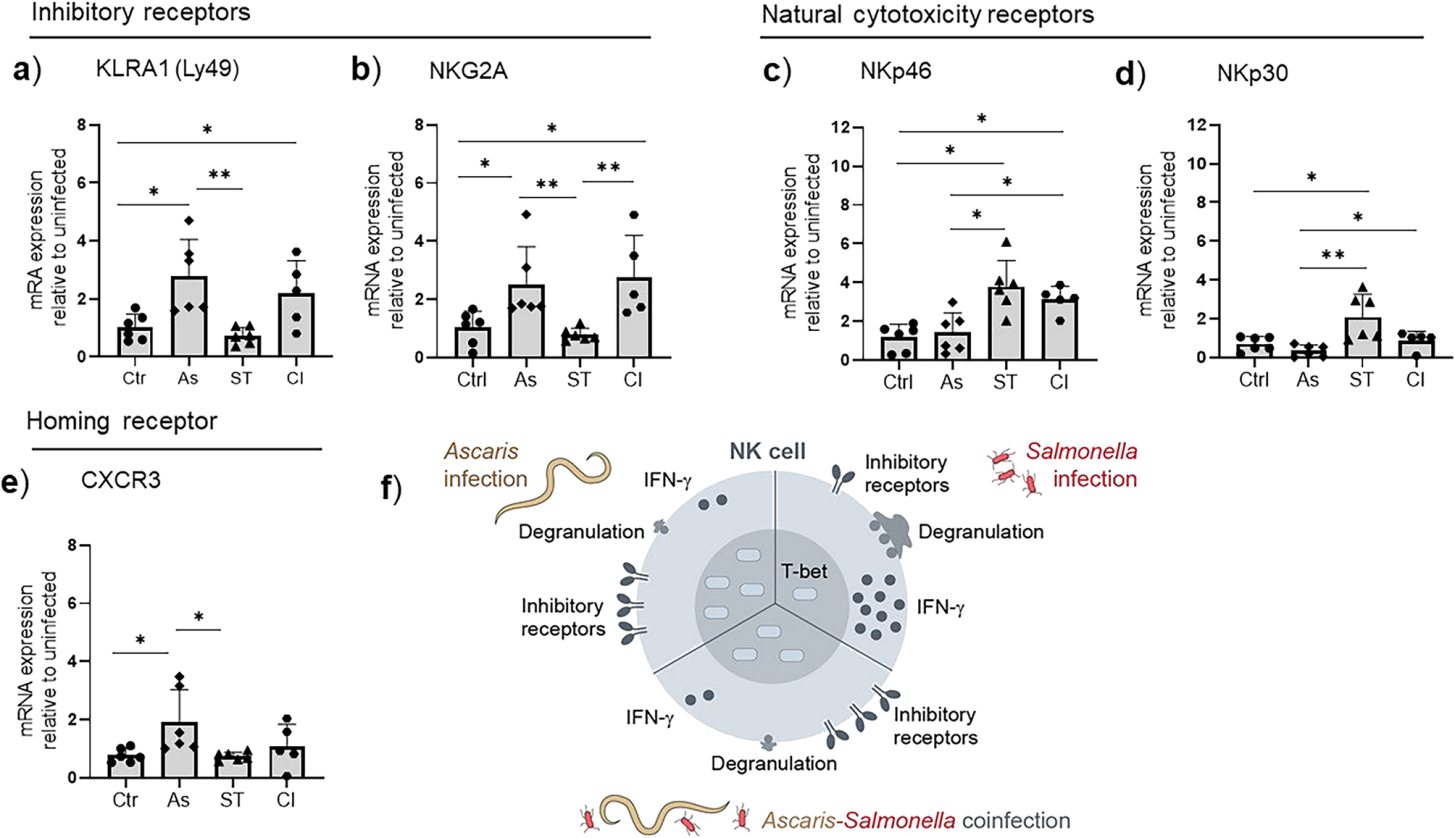
*Ascaris* induces upregulation of the killer inhibitory receptor genes in FSC/SSC^low^ CD3-CD16 + CD8α NK cells during *Ascaris*–*Salmonella* coinfection. Relative gene expression of (**a**) KLRA1 (Ly49), (**b**) NKG2A, (**c**) NKp46, (**d**) NKp30, and (**e**) CXCR3 in the FACS-sorted NK cells from the spleen of the control (n = 6), *Ascaris-*infected (n = 6), *Salmonella* (n = 6) and *Ascaris–Salmonella* coinfected pigs (n = 5, one sample was excluded from analysis due to low quality). Controls (Ctr), *Ascaris* (As), *Salmonella* (ST), and coinfection (CI). Significant mean differences between the groups are indicated (ANOVA test, *p < 0.05, **p < 0.01). (**f**) Graphical illustration shows suppression of NK cells’ IFN-γ production and degranulation capacity during *Ascaris*-single infection and *Ascaris*–*Salmonella* coinfections in domestic pigs. This suppression is associated with the upregulation of T-bet transcription factor and NK cell inhibitory receptors.

The activation and regulation of NK cell function depends largely on the activating and inhibitory receptors^32^. Considering that T-bet overexpression is associated with terminal differentiation and upregulation of inhibitory receptors, we determined gene expression levels of inhibitory receptors (KLRA1/Ly49 and NKG2A) which facilitates MHCI interactions and NK functionality^30^. In addition, we evaluated gene expression of the natural cytotoxicity receptors (NCR) (NKp46 and NKp30) which are readily expressed upon EOMES overexpression^30^. Based on the mRNA profiles determined with FACS-sorted splenic NK cells from the different groups, we found that NK cells from *Ascaris*-single infected and *Ascaris*–*Salmonella* coinfected pigs were marked by the significantly upregulated expression of NK cell inhibitory receptors compared to uninfected controls and *Salmonella*-single infected groups (Fig. 5a,b). There was no upregulation of NCR genes in the *Ascaris* group though we noticed minimal but significant upregulation of NKp46 and NKp30 in the *Ascaris-Salmonella* coinfected group (p = 0.01) (Fig. 5c,d). The upregulation of NCRs in the coinfection group without the matching increase in functionality may be attributed to the counter effects of NK cell inhibitory receptors overexpression by *Ascaris* infection*.* In addition, we determined CXCR3 chemokine gene expression on the sorted splenic NK cells since expression of CXCR3 is associated with T-bet expression and cell trafficking in murine models^33^. Significantly higher expression of CXCR3 genes (p = 0.03) was observed in the *Ascaris* group compared to the controls indicating better homing capacity and possible higher interaction with T-bet. Overall, our results therefore suggest that *Ascaris* hinders the effector functionality of NK cells through the upregulation of NK cell inhibitory receptors.

## Discussion

*Ascaris* infections occur predominantly in developing countries which often lack adequate sanitation thereby bearing a higher risk of coinfection with other microbial pathogens such as *Salmonella*^34,35^. As a major cause of foodborne diseases in humans, *Salmonella* infections pose a huge public health problem globally with chicken and pigs representing the main contaminated meat source^36^. The emergence of infections with multi-drug resistant *Salmonella* in addition to the lack of an optimal vaccine calls for a better understanding of the host immunity^37^. This gains further relevance in the context of helminth coinfections which can antagonize the host's protective responses against *Salmonella* and other intracellular pathogens^7^. Domestic pigs not only serve as an important meat source but also as important carriers of zoonotic *A. suum* and *Salmonella* infections^6,9,38^. The domestic pig thus offers a valuable model for translational research as part of a one health approach.

The severity of *Salmonella* infections is dependent on the infecting serovar, the infection dose, age, and the immunological status of the host^39^. The subspecies *Salmonella enterica* serovar Typhimurium is a major cause of clinical diseases both in humans and animals. To date, little is known about the role of NK cells in helminth and bacterial co-infections both in humans and pigs. In the murine model, NK cells plays a prominent role in the control of *Salmonella* infections during the early stages^16,40,41^. Furthermore, helminth-*salmonella* coinfection in mice leads to impaired host resistance against *Salmonella*^14^. We have previously demonstrated in vitro modulation of NK cells and dendritic cells by *Ascaris* excretory-secretory products^19^. Here we aimed to gain more insight into the possible immunomodulatory effects of *Ascaris* on NK cells during coinfection with *Salmonella* in domestic pigs, which are their natural host.

Our results show that acute *Ascaris* infections significantly compromise the capacity of NK cells to produce IFN-γ in pigs. This *Ascaris*-induced modulatory effect was similarly evident in the *Ascaris*–*Salmonella* coinfection group. In support of our findings, both in murine models and in vitro experiments, NK cells rapidly produce IFN-γ in response to *Salmonella* infection^16,17,42^. In addition, among other immune cells, NK cells are the largest population of IFN-γ producers in response to IL-12 and IL-18 stimulation^16^. Following the breaching of the epithelial barrier, *Salmonella* preferentially invades gut resident macrophages which respond by activating the neighboring NK cells both by direct cell-to-cell contact and through the release of IL-12 and IL-18 cytokines^43^. Activated NK cells in turn produce IFN-γ which further activates the infected macrophages^16^. IFN-γ enhances the release of reactive oxygen and nitrogen species, hydrolytic enzymes and antimicrobial peptides leading to phagolysosome enrichment and subsequent killing of the intracellular bacteria^44^. Deficiencies in the IL-12/IFN-γ axis in mice and humans increase susceptibility to *Salmonella* infections through impaired granuloma formation, resulting in uncontrolled bacteria growth inside the macrophages^45,46^. Thus, inadequate IFN-γ production in *Salmonella* infections as a result of *Ascaris* infection as seen in the current study would potentially antagonize the protective host immunity against *Salmonella*. In concordance with the *Ascaris*-induced impairment of NK cell functionality, the coinfected pigs had higher live *Salmonella* burdens compared to the *Salmonella*-single-infected group^15^, though the mechanistic link with NK cells functionality calls for further investigation.

We show that *Ascaris* potentially suppresses the cytotoxic function of NK cells as exhibited by the significantly lower surface expression of the degranulation marker CD107a (LAMP-1). Furthermore, the NK cells' degranulation potential was impaired in the coinfection setting which was coupled with strongly reduced levels of perforin compared to the *Salmonella*-single group. In mice, IL-18-mediated NK cellular cytotoxicity through perforin responses is crucial for resistance against salmonellosis during the early stages of infection^28^. The hyporesponsiveness to IL-12 and IL-18 stimulation for both cytotoxicity responses and IFN-γ production observed in the *Ascaris-*single infection and coinfection implies that *Ascaris* clearly impedes the functionality of NK cells.

Whether NK cells exert direct immunological control over the *Ascaris* larvae remains unkown. Of note, the depletion of murine NK cells early during infection with the gastrointestinal nematode *Heligmosomoides polygyrus* infection did not affect the worm burden but rather resulted in increased endothelial injury^47^. This may suggest that *Ascaris* also modulates the effector functions of NK cells in its natural hosts either to avoid larval killing, possibly through the repression of NK cell cytotoxic functions, or to benefit from NK activity associated tissue homeostasis and wound healing. However, this complex interaction remains to be elucidated. The chemokine receptor CXCR3 (CD183) is associated with T-bet and the recruitment and trafficking of murine immune cells^33^. Thus, the upregulation of CXCR3 genes to possibly increase NK cell trafficking capability by acute *Ascaris* infection without the induction of cytokine production and cytotoxicity function suggests that NK cells are still required for other functions such as immune regulation and/or wound healing to promote disease tolerance.

We show that *Ascaris* modulates the functionality of NK cells by upregulating the expression of the T-bet transcription factor. Murine NK cell development, maturation, and functionality is orchestrated by the balances between EOMES and T-bet transcription factors^31^. Whilst EOMES is dominantly expressed in immature NK cells, T-bet is specifically essential for the terminal differentiation of NK cells and epigenetically promotes the expression of NK cell inhibitory receptors expression^30^. This is in agreement with the findings of this study whereby the induction of terminally differentiated NK cells by *Ascaris* was significantly associated with (Ly49/KLRA1, and NKG2A/KLRD1) inhibitory receptors gene expression. Thus, *Ascaris*-induced impairment of NK cells may be attributed to the upregulation of NK cell inhibitory receptors which are key checkpoints inhibitors controlling the activation of NK cells.

The natural cytotoxicity receptors NKp46 (CD335), NKp44 (CD336), and NKp30 (CD337) are essential for NK cell degranulation and cytokine production^32^. Although the majority of porcine NK cells are NKp46 negative, CD8α^dim^NKp46^high^ NK cells are highly activated possessing a higher IFN-γ producing capacity as well as the enhanced ability to release cytolytic granules following IL-12 and IL-18 stimulation^21,48^. Accordingly, CD8α^dim^ NK cells showed the highest IFN-γ production capacity in the current study. Thus, the downregulation of the activating receptor genes by *Ascaris* together with the upregulation of inhibitory receptor genes would hinder both the cytokine and degranulation capacity of the NK cells as observed here. This study was limited to assess NKp46 expression in NK cells.

In conclusion, our data show that NK cell functionality is impeded by acute *Ascaris* infection in pigs. This *Ascaris*-associated impairment of NK cells was also evident in the *Ascaris-Salmonella* coinfection setting. Further elucidation of the effects of the acute and also chronic *Ascaris* infections on NK cells in promoting susceptibility, bacterial burdens and severity of *Salmonella* infection in pigs and/or in humans will be of uttermost importance. In addition, with the evolution of memory in NK cells^49^, evaluation of the longevity and mechanisms of the NK cell reprogramming by *Ascaris* would be essential as NK cells are crucial for intracellular pathogen and tumor control.

## Methods

### Ethics statement

Ethical approval was obtained from the State Office of Health and Social Affairs Berlin, Germany (Landesamt für Gesundheit und Soziales Berlin, Germany) (G0212/20). The study was performed following the European Convention for the Protection of Vertebrate Animals used for Experimental and other Scientific Purposes and the German Animal Welfare Law. This study is reported in accordance with ARRIVE guidelines 2.0.

### *Ascaris suum* and *Salmonella enteritica* infection

24 German landrace hybrid pigs (12 males and 12 females) from a conventional breeding facility (Brandenburg district, Germany) at 6–7 weeks of age, were used for the experimental infections. A"trickle-like" *Ascaris* infection regimen was adopted from previously reported doses (< 10,000 eggs) to mimick a ‘natural’ exposure setting^50^. A sub-clinical dose was used for *Salmonella* infections^51^. 6 pigs (*Ascaris* group) were orally infected with 2000 embryonated *Ascaris suum* eggs on 4 consecutive days (total inoculation dose of 8000 eggs, embryonation rate 97.80%). *Ascaris suum* egg collection, density purification and evaluation of the embryonation rate were carried out as previously reported^52^. The *Ascaris* group pigs were dissected two weeks post-infection. The *Salmonella* group entailed 6 pigs that were orally infected with *Salmonella* Typhimurium definitive type 104 with a single inoculum dose of 10^7^ colony-forming units (CFU) and dissected one week post-infection. Similarly, the *Ascaris-Salmonella* coinfection group (n = 6) was orally infected with 2000 embryonated *Ascaris suum* eggs on 4 consecutive days (total inoculation dose of 8000 eggs), and co-infected with *Salmonella enterica* serovar Typhimurium (inoculum dose of approximately 10^7^ CFU) one week after *Ascaris* infection followed by dissection one week later. The pigs were kept for 14 days to allow them to acclimatize before the experimental infections. During this period, the pigs were also thoroughly examined for any pre-existing helminth or *Salmonella* infections. All pig groups, including the uninfected group (n = 6), were kept in separate indoor pens with a controlled light system. In addition, the stables were equipped with a centrally controlled heating system. The pigs received a standard diet based on their body weight, which was determined once a week and water was given ad libitum.

### Blood collection and isolation of porcine peripheral blood mononuclear cells

Blood was collected in EDTA tubes (S-Monovette, Sarstedt) by heart puncture after sedation with ketamine hydrochloride (20 mg/kg BW, Ursotamin, Serumwerk Bernburg AG, and 2 mg/kg BW), azaparone (Stresnil, Janssen-Cilag GmbH, Germany) and xylazine (36 mg/kg BW; Xylavet; CP-Pharma Handels GmbH). Peripheral blood mononuclear cells (PBMC) were isolated from whole blood by density gradient centrifugation using 0.9% sodium chloride solution at a 1:1 ratio using pancoll (1.077 g/ml density; PAN-Biotech GmbH; Aidenbach, Germany) using Leucosep tubes (Greiner Bio-One, Austria). Following the density gradient centrifugation at 1500 rpm for 20 min at room temperature (RT), PBMC were carefully obtained at the interface and washed in 0.9% sodium chloride. The remaining erythrocytes were lysed using erythrocyte lysis solution (H_2_O plus 0.01 M KHCO_3_, 0.155 M NH_4_Cl, and 0.1 M EDTA, pH 7.5) at RT for 5 min. PBMC were thereafter washed twice and resuspended in complete Iscove’s modified Dulbecco’s medium (cIMDM; IMDM with stable glutamine, 25 nM HEPES and 3.024 g/l NaHCO3, plus 10% fetal bovine serum (FBS), 100 U/ml penicillin and 100 μg/ml streptomycin; PAN-Biotech GmbH; Aidenbach, Germany).

### Isolation of splenocytes

Pooled tissue sections from the spleen were sampled following euthanization of the pigs by intracardial injection of tetracaine hydrochloride, mebezoniom iodide, and embutramide (10 mg/kg BW; T61, Intervet, Germany). The spleen sections were mechanically disrupted and the suspension passed through a 70 µm cell strainer. Washing, erythrocyte lysis and pellet resuspension were carried out as described for the PBMC isolation.

### Stimulation of NK cells with IL-12 and IL-18

Freshly isolated PBMC and splenocytes were counted using the CASY automated cell counter and cultivated in cIMDM at a density of 1 × 10^6^ cells/ml in a final volume of 200 μl. Cells were stimulated with either 25 ng/ml porcine IL-12p70 and 100 ng/ml porcine IL-18 (R&D Systems; Minneapolis, USA) or left in the culture medium alone as negative control and incubated overnight at 37 °C and 5% CO_2_. Brefeldin A (ThermoFisher; Waltham, MA, USA) was added after 2 h of stimulation at a final concentration of 3 μg/ml. The NK cells degranulation capacity was assessed through the surface expression of CD107a following IL12/18 stimulation. During the addition of Brefeldin A, the cultures were supplemented with Alexa Flour® 647 conjugated mouse anti-pig CD107a antibody (IgG1, clone 4E9/11, Bio-Rad).

### Flow cytometry analysis

Before flow cytometry (FCM) staining for the analysis of NK cells both the stimulated and unstimulated PBMC and spleen cells were resuspended in PBS (PAN-Biotech GmbH; Aidenbach, BY, Germany) containing 5% (v/v) FCS and 2 mM EDTA. Mouse serum (1:500) was used for blocking prior to staining. Surface markers (CD3, CD8α and CD16), cytokines (IFN-γ and TNF-α), transcription factors (EOMES and T-bet) and perforin were stained using pig-specific or cross-reactive antibodies outlined in (Supplementary Table 1). The fixable viability dye eFluor® 506 (ThermoFisher; Waltham, MA, USA) was used for dead cell exclusion. Cells were fixed and permeabilized with a Transcription Factor Staining Buffer Set (eBiosciences) to facilitate intranuclear staining. All incubation steps were performed for 10 min at 4 °C. The FCM analysis of NK cells was performed on BD FACSAriaIII (BD Biosciences). FCM data was acquired using the FACSDiva software (BD Biosciences) and evaluated using FlowJo software (version 10.0, Tree Star, Ashland, OR, USA).

FACS sorting of CD3-CD8α + CD16 + NK cells from spleen cells was performed on BD FACSAriaIII (BD Biosciences). In addition, eFluor® 506 fixability dye was included to discriminate between the live and dead cells. The average purity of the sorted NK cells was 95.0%. The NK cells were directly sorted in RNAlater® (Sigma-Adrich, Germany) and stored at − 80 °C for further gene expression analysis.

### Evaluation of gene expression by quantitative reverse transcriptase PCR

Total RNA from the sorted NK cells was extracted using the RNeasy Mini kit (Qiagen, Germany) following the manufacturer’s protocol. The amount and the purity of the extracted RNA was determined using a Nanodrop ND2000 spectrophotometer (Nanodrop Technologies) at 260/280 nm. Gene expression from the unstimulated NK cells was evaluated using the Light-Cycler® 480 II system (Roche) following total RNA transcription into cDNA using the High Capacity RNA-to-cDNA kit (Applied Biosystems). SYBR® Green I Master Mix (Roche) was used for the amplification of the target genes. Internal standard from equal pooled cDNA of all the samples analysed in this study was used for standard curve generation for subsequent gene expression evaluations. Primers used for all gene targets were adopted from^48,53^, and all primers were commercially synthesised (TIB-Molbiol, Germany). Sequences for all primers are provided in (Supplementary Table 2). To normalize target-gene expression the housekeeping genes of ribosomal protein L19 (RPL19) and glycerinaldehyd-3-phosphat-dehydrogenase (GAPDH) were used. Samples were analysed in duplicate. Relative expression of the target genes was calculated using the efficiency-corrected 2^−ΔΔCT^ method^54^.

### Statistical analysis

Statistical analysis was done using GraphPad PRISM version 9.0 (GraphPad Software, USA). Normality was tested with the Shapiro–Wilk test. One-way variance analysis (ANOVA) with Bonferroni correction for paired sample means was applied,* p* < 0.05 was considered significant.

### Supplementary Information

Supplementary Information.

## Data Availability

The datasets generated during and/or analysed during the current study are available from the corresponding author on reasonable request.
