# Supplementary material for: Acute Ascaris infection impairs the effector functions of natural killer cells in single and Salmonella co-infected pigs
Source: Sci Rep. 2024 Jun 25;14:14586. doi: 10.1038/s41598-024-64497-4 (PMC11199589; doi:10.1038/s41598-024-64497-4)
Supplement: Supplementary file 1 — Supplementary Information. [file 41598_2024_64497_MOESM1_ESM.pdf]

Acute *Ascaris* infection impairs the effector functions of natural killer cells in single and *Salmonella* co-infected pigs

Robert M. Mugo[1], Larissa Oser[1], Ankur Midha[1], Joshua Adjah[1], Arkadi Kundik[1], Alexandra Laubschat[1], Philipp Höfler[1], Zaneta D. Musimbi[1], Rima Hayani[1], Josephine Schlosser-Brandenburg[1], Susanne Hartmann[1], *Sebastian Rausch[1]

Affiliations

[1]Institute of Immunology, Centre for Infection Medicine, Freie Universität Berlin, Berlin, Germany

Supplementary Information

Supplementary Table 1: List of the antibodies used for flow cytometry analysis

	Clone	Reactive species	Isotype	Dilution	Fluorochrome	Source company
CD8a	76-2-11	Pig	IgG2a	1:100	Purified- (Rat-anti mouse Ig2a PE-Cy7-)	Biolegend
CD16	G7	Pig	IgG1	1:20	FITC	Bio-Rad
CD107a	4E9/11	Pig	IgG1	1:20	A647	Bio-Rad
CD3	BB23-8E6-8C8	Pig	IgG2a	1:100	Streptavidin-Alexa700	ThermoFisher
IFN-γ	P2G10	Pig	IgG1	1:100	PerCp-Cy5.5	ThermoFisher
TNF-α	Mab11	Human (Cross-reactive to pig)	IgG1	1:100	APC-Cy7	Biolegend
Perforin	δG9	Human (Cross-reactive to pig)	IgG2b	1:50	efluor 450	ThermoFisher
T-bet	4B10	Human (Cross-reactive to pig)	IgG1	1:100	BV605	Violet A
EOMES	WD1928	Human (Cross-reactive to pig)	IgG1	1:50	PE	ThermoFisher

Supplementary Table 2: Primers used for the real-time quantitative reverse transcriptase polymerase chain reaction of sorted porcine NK cells.

Accession number	Target	Sequence
EU282355.1	NKp30	**F**: TCTATTACCAGGGCAAATGTGAAGT
		**R:** GTCACTGGGGTCTAGAATCACTCAT
NM_001123143	NKp46	**F**: CCTTTGACCAGGAGCTTCACA
		**R:** AGATGTTTTGGCTCCTACAACAAG
XM_003135179.3	CXCR3	**F:** CCGACCACAAGCACCAAAGCA
		**R:** TGGCGTTGGCTCATCTCAGGGA
XM_021091173.1	KLRA1 (Ly49)	**F**: TGGCAAGACTGATGAAAAAGAGTT
		**R:** GAAACAGAGGATCCCAAGAATCAC
XM_021092358.1	KLRC1 (NKG2A)	**F:** GTAATTGTCCAAAGGAATGGTTTACA
		**R:** CGAAGTAGAGTAGAATTCCGTGAAGC

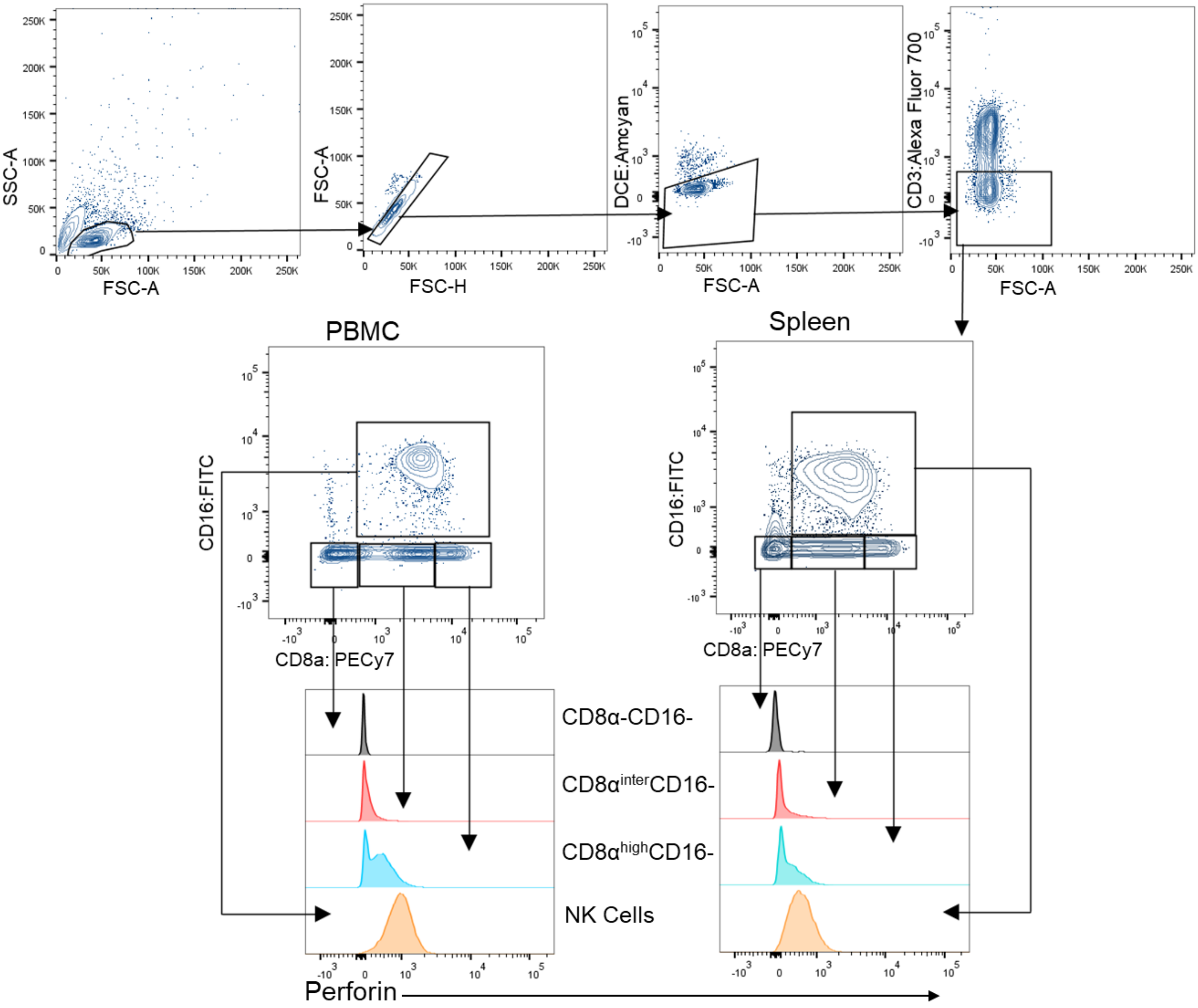

Supplementary Figure 1: Gating strategy used for the phenotyping of porcine NK cells. Lymphocytes from PBMC and spleen were gated from the forward scatter-area (size) (FSC-A^{low}) and side scatter-area (granularity) (SSC-A^{low}), and singlets were subsequently selected using forward scatter-area and height. Dead cells and debris were excluded using a fixable viability dye in eFluor® 506. NK cells were further identified as CD3-CD16+CD8α+perforin+ cells.

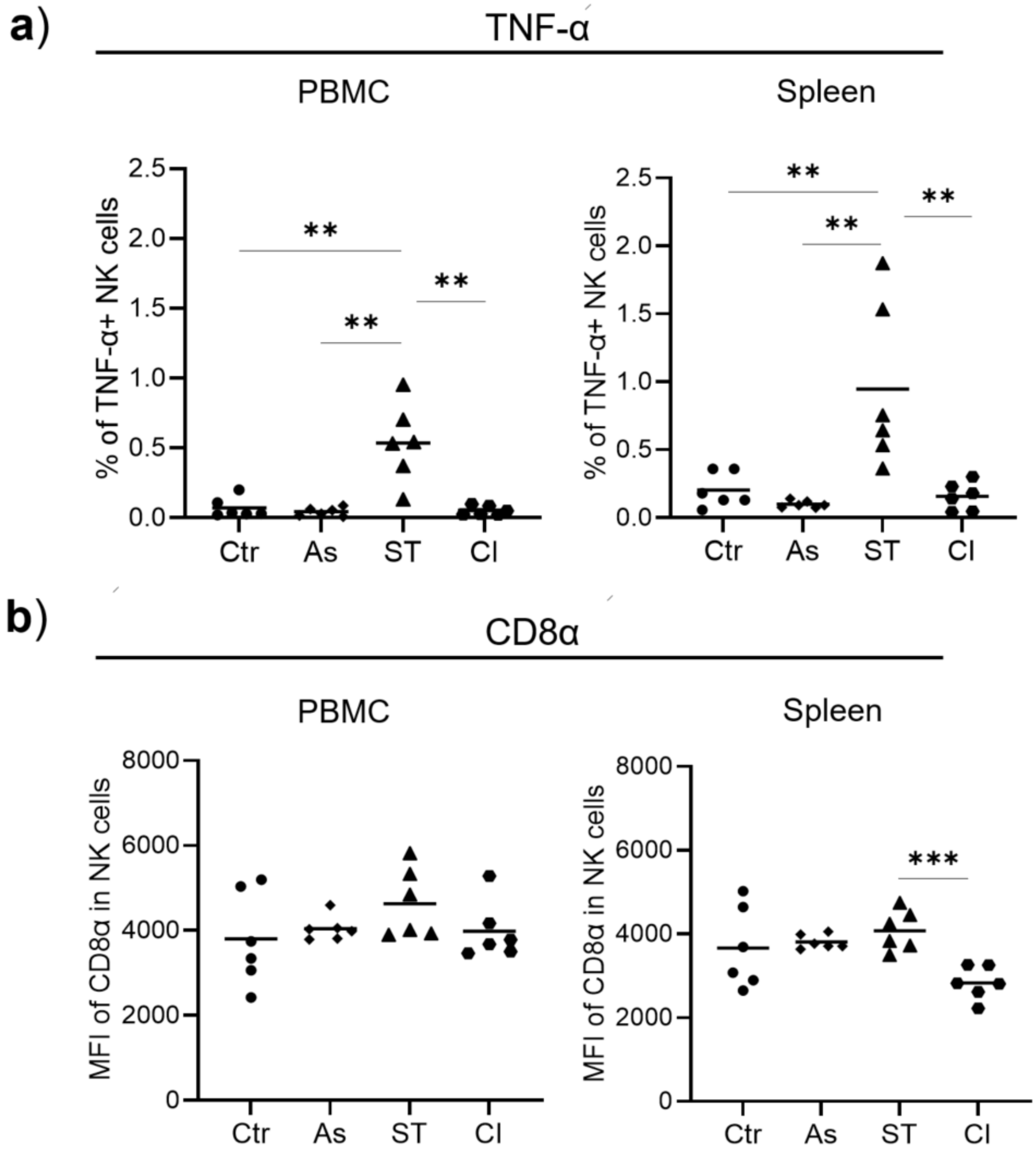

Supplementary Figure 2*:* TNF-α production and CD8α expression by NK cells in *Ascaris* and *Salmonella*-single infection and *Ascaris*-*Salmonella* coinfection. **a**) Percentage of TNF-α positive FSC/SSC^{low} CD3-CD16+CD8α+Perforin+ NK cells in PBMC and spleen (n=6 for all groups). **b**) Comparisons of the CD8α mean fluorescence intensity of blood and spleen NK cells (n=6 per group). Controls (Ctr), *Ascaris* (As), *Salmonella* (ST), and coinfection (CI). Significant mean differences between the groups in blood or spleen are indicated (ANOVA test, ** = p < 0.01, *** = p < 0.001).

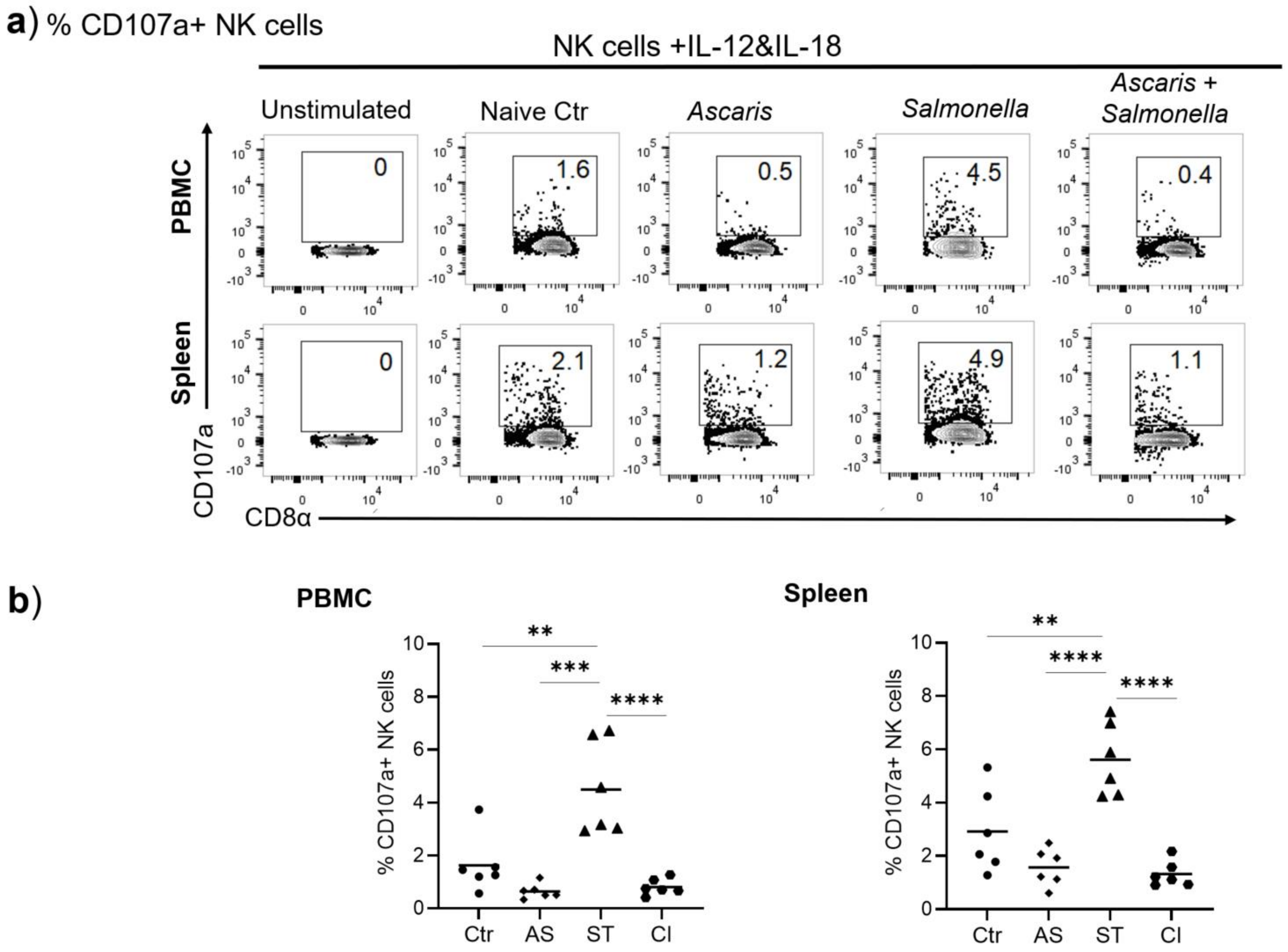

Supplementary Figure 3: Impaired cytolytic potential of FSC/SSC^{low} CD3-CD16+CD8α+Perforin+NK cells in *Ascaris*-*Salmonella* coinfection. **a)** Representative flow cytometry contour plots showing the percentages of CD107a expressing blood and splenic porcine NK cells of the control, *Ascaris*-infected and *Ascaris*-*Salmonella* coinfected pigs following stimulation with recombinant IL-12 and IL-18. **b)** Percentages of CD107a positive NK cells in PBMC and spleen (n=6 for all groups). Controls (Ctr), *Ascaris* (As), *Salmonella* (ST) and coinfeciton (CI). Significant mean differences between the groups in blood or spleen are indicated (ANOVA test, ** = $p < 0.01$, *** = $p < 0.001$, **** = $p < 0.0001$).
